# The effectiveness of the STOP-X training program on the knee valgus angle and balance in female basketball players with dynamic knee valgus: a randomized controlled trial

**DOI:** 10.1186/s13102-024-00844-2

**Published:** 2024-02-21

**Authors:** Mohadeseh Rostami, Parisa Sedaghati, Hassan Daneshmandi

**Affiliations:** Department of Sport Injury and Corrective Exercise, Faculty of Sport Sciences, University of Guilan, Rasht, Iran

**Keywords:** Knee valgus, Anterior cruciate ligament, Injury prevention, STOP-X, Basketball

## Abstract

**Background:**

Dynamic knee valgus (DKV) accompanied by poor balance is the cause of anterior cruciate ligament (ACL) injury in athletes, and the identification and correction of these factors are always of interest to researchers. Therefore, the purpose of this research was to investigate the effect of the STOP-X program on the knee valgus angle and static and dynamic balance in female basketball players with DKV defects.

**Methods:**

The present study was a quasi-experimental study. Thirty female basketball players with DKV defects were purposefully identified by the single-leg landing (SLL) test and were randomly assigned to two control (*n* = 15) and experimental (*n* = 15) groups. Static balance status was evaluated with the BASS STICK test, and dynamic balance status was evaluated with the Y-balance test (YBT). The experimental group performed the STOP-X program for 25–40 min for eight weeks (three times per week), and the control group performed their traditional warm-up program. Data were analyzed by means of 2 × 2 repeated measures ANOVA followed by post hoc comparison (Bonferroni) at the significance level of (*P* < 0.05) with SPSS version 26.

**Results:**

The results showed that with the use of the STOP-X program, there was a significant difference between the experimental and control groups in variables of the static balance (F = 56.45; *P* = 0.001; ES = 0.66, PC=↑59.64%), total dynamic balance score (F = 107.57; *P* = 0.001; ES=↑0.79, PC=↑19.84%), and knee valgus angle (F = 119.46; *P* = 0.001; ES = 0.81, PC=↓34.36%).

**Conclusion:**

In addition to reducing the knee valgus angle, applying the STOP-X injury prevention program can improve static and dynamic balance in female basketball players with DKV defects. Therefore, it can be recommended that sports trainers benefit from these advantages by adding STOP-X training to routine basketball exercises.

## Introduction

Anterior cruciate ligament (ACL) injuries are among the most common injuries in sports, specifically basketball. Female athletes aged 14–19 years have the highest incidence of ACL injury [1, 2]. These injuries have, in part, been associated with the nature of the sport, which involves high-speed changes in direction, jump landing, and pivoting, but more specifically, with the particular movement strategies adopted by these female athletes [3]. Levine et al. reported that combinations of anterior tibial shear force, knee abduction, and internal tibial rotation moments may lead to ACL failure [4]. Reportedly, female basketball players adopt more valgus positions on landing than female athletes from other sports, which may, in part, explain their increased risk [5].

Otherwise, studies have shown that individuals with poorer dynamic balance and core stability are more likely to experience lower extremity injuries [6, 7]. Therefore, poor balance is one of the leading causes of injury, especially in the lower extremities. When the knee assumes a valgus position during an activity, the joint is placed in a less stable position and prone to injury [8]. Accordingly, valgus loading reduction is an effective strategy for reducing the incidence of ACL injuries. Therefore, surgery and rehabilitation prevent adolescents from participating in sports activities for a long period, affecting healthy physical development. In addition, regardless of management, the risk of developing osteoarthritis significantly increases after an ACL injury [9, 10]. Thus, adopting urgent measures to correct this abnormality before reaching pathology can be helpful, indicating the need to develop injury prevention programs. Consequently, given the importance of warm-up and specific exercises for athletes with ACL injuries, the German Knee Society (DKG) has presented a specific training program called STOP-X for the knee joint. According to the name of this program (STOP-X), it aims to reduce the knee valgus angle (X-shaped position), which is one of the important risk factors in knee injuries, especially ACL injury. Also, in addition to correcting dangerous movement patterns, this program offers strategies to improve balance, as well as neuro-muscular training for inter- and intra-muscular optimization and hip stabilization exercises. It has been reported that this training program can reduce the incidence of knee injuries by up to 27% and ACL injuries by up to 51% [11].

In this respect, Herrington et al. reported that the drop jump knee valgus angle in the left leg on average was reduced by 9.8°, and that in the right leg was reduced by 12.3°; during the jump shot, the knee valgus angle in the left leg showed a mean reduction of 4.5°, and that in the right leg was reduced by 4.3° following 4 weeks of plyometric training on female basketball players [12]. Additionally, Kato et al. reported an improvement from 36.9° to 15.1° in the coronal-plane lower-extremity maximal angles and from 22.5° to 17.1° in the transverse (horizontal)-plane torsion angle following a course of neuromuscular exercises on basketball players [13]. Otsuki et al. evaluated the effect of injury prevention training (including warm-up) on dynamic knee alignment in female basketball players. According to the results, knee valgus motion and the probability of a high knee abduction moment (pKAM) did not significantly differ between the experimental group and the control group, but did increase [14]. In addition, Eslami et al. explored the effect of 8 weeks of STOP-X and FIFA 11 + Kids Warm-Up intervention on proprioception and balance in soccer players with DKV. The study findings suggested that STOP-X exercises are more effective than FIFA 11 + exercises at improving DKV and balance among young football players with knee valgus abnormalities [15].

Otherwise, basketball is a sport that places significant demands on the knee, requiring running, jumping, and cutting movements that can put the players at risk for ACL injuries [16]. Since females are more prone to ACL injuries than males due to an increased knee valgus angle during landing [17], preventing ACL injury is of paramount importance for basketball players, especially female basketball players. Also, all the previous studies of STOP-X have been done on men, however, a literature review has shown that no research has been conducted on the effectiveness of STOP-X training on women who demonstrate hormonal, anatomical, and neuro-muscular differences compared to men [18]. Also, the functional test and dynamic valgus evaluation method are not the same in all of them. Since most non-contact ACL injuries occur as a result of landing or decelerating on a limb [19], the test we used to assess dynamic knee valgus was the SLL test because of its similarity to the mechanism of ACL injury, especially in basketball players. Therefore, this study investigated the effect of the STOP-X injury prevention program on the knee valgus angle and static and dynamic balance in female basketball players with DKV defects.

## Materials and methods

### Study design

The present study was a quasi-experimental design due to the presence of the control group and also an intervention in the experimental group.

### Participants

The population included 30 female basketball players (*n* = 30, age = 15.50 ± 1.52 years, height = 1.62 ± 0.06 cm, weight = 55.15 ± 9.65 kg, BMI = 20.69 ± 2.80 kg/m^2^_,_ leg length = 85.46 ± 3.69 cm, sports history = 3.73 ± 1.56 years) with DKV defects. The sample size was determined to be 30 by purposive sampling using G*Power software with a desired power of 0.50, an alpha of 0.05, and an effect size of 0.75. This effect size was comparable to previous research reporting changes in landing mechanics after neuromuscular training with dual cognitive tasks [20].

Randomization.

Randomization was performed by an independent investigator unfamiliar with the testing protocol using a random allocation rule. The letters A and B were identified as markers for random groups assignment and were placed in sealed opaque envelopes in a box. Another researcher opened envelopes and proceeded with training according to the group assignment. These letters were numbered randomly selected and placed one after the other. Thus, the subjects were divided into two groups A (experimental = 15) and B (control = 15). Group allocation was concealed using an opaque envelope until after athletes had been enrolled in the study to minimize potential bias.

Furthermore, the present study was a single-blind study in which only the researchers knew which intervention the participants were receiving and the subjects did not know which study group they were in until the study was over. The pre-test lasted for 1 week. To prevent injury, the subjects were asked to perform an initial warm-up before doing the pre-test. After the pre-test, the experimental group underwent STOP-X training, in addition to their usual training. In that time, the subjects of the control group performed their usual exercises. After 8 weeks, the post-test was conducted, which lasted for 1 week. Afterwards, the collected data were analyzed.

Eligibility criteria.

**Fig. 1:**
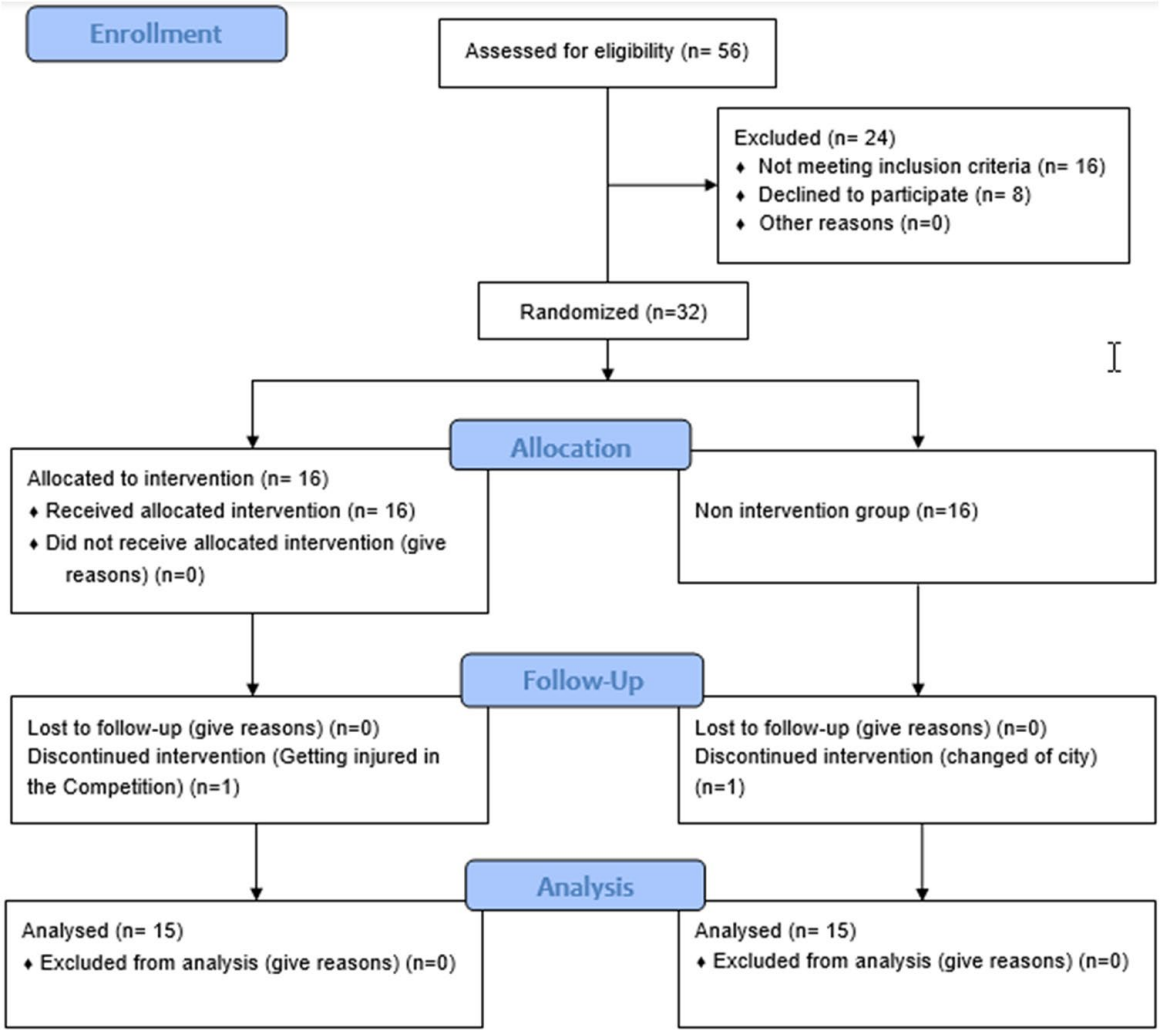
Study flowchart

The inclusion criteria consisted of 14- to 19-year-old females with a knee valgus angle greater than 12° during single-leg landing (SLL) [21, 22], a minimum of 3 years of training experience, a regular training routine (at least three training sessions per week), no history of lower extremity injuries in the past six months leading to functional or structural limitations or lower extremity surgery leading to changes in normal alignment, and the absence of spine-related and upper extremity musculoskeletal disorders. Otherwise, the exclusion criteria consisted of noncooperative subjects in the pretest or posttest, failure to complete the training program for any reason, absence of more than three exercise sessions or two consecutive sessions [23], pain or injury during exercise, and unwillingness to continue cooperation. This research was performed under controlled and identical conditions for subjects in a safe environment at the Qazvin Basketball Club. Once selected, the participants signed an informed written consent form, including explanations about the steps, objectives, and research participation conditions. Ethical considerations were followed in this research, and the code of ethics (ID IR.GUILAN.REC.1402.015) was obtained from the Ethics Committee in Biomedical Research (ETHICS), Guilan University. This study adhered to CONSORT guidelines for randomized controlled trials and had been registered in the Iranian Registry of Clinical Trials (ID: IRCT20231230060574N1, on 04/01/2024). subjects’ allocation and dropouts were remarked at the study flowchart (Fig. 1).

### Outcome measurement

Single-leg landing test.

This study screened and determined the DKV using the SLL test, which demonstrated a reliability of 0.87 [24]. To perform this test, the subjects dropped directly down off a 30-cm-high box onto a mark 30 cm from the box, made landfall on the dominant leg and held the position. The participants then stood in a balanced position (stance) near the edge of the box so that the dominant leg was suspended (heel in contact with the edge of the box). A digital video camera (with external memory) was placed on a tripod at the height of the subject’s knee at a distance of 230 cm from the box in the frontal view. Three successful trials were recorded for each subject. The mean angles of the three trials above were used in the final analysis. The knee valgus angle of the dominant leg was then measured using the Kinovea software package (reliability = 0.98) [25]. A frame-by-frame analysis of the video images demonstrated that the complete landing image is the frame where the subject is at the lowest height (i.e., maximum knee flexion). The angle subtended between the lines formed between the markers at the ASIS and middle of the tibiofemoral joint and that formed from the markers on the middle of the tibiofemoral joint to the middle of the ankle mortise was recorded as the valgus angle of the knees [21, 26]. The identified markers were located in three areas: the ASIS of the landing leg, the center of the patella, and the center of the ankle mortise. The normal knee valgus angle was in the range of 5–12° for females during the SLL test [21, 22]. Females with knee valgus angles greater than 12° during SLL were included in this study as those with DKV. Harrington et al. suggested that 2D video kinematics have a reasonable association with what is being measured with 3D motion capture [24].

Static balance test.

Static balance was evaluated using the Bass–Stick test, which records how long a person can stand for 60 s on a 2.5-cm wooden block without touching the ground. This test was performed three times on the dominant leg, and the best result was considered the static balance test score [27]. Test-retest reliability was reported to be 0.91 [28].

Dynamic balance test.

To perform the dynamic balance test, the true leg length (from the ASIS to the distal portion of the medial malleolus) was measured to normalize the data and compare the subjects. Additionally, the dominant leg was evaluated using the ball-kicking test. This research employed the Y-balance test (YBT) to evaluate dynamic balance. Y-Balance Test Kit was also used to evaluate this test. Before the test, each subject performed the test three times as a practice to minimize the learning effect. After that, the subject rested and performed the test three times with dominant leg. The participants were asked to stand in the center of their directions, place themselves on the dominant leg, reach the other leg, return to the normal position on both legs, and maintain their position for 10–15 s before the next trial. All trials in a single direction had to be completed sequentially clockwise or counterclockwise before proceeding in the next direction. The subjects were asked to touch the farthest possible point in any given direction with their toe. The reach distance was defined as the distance from the contact point to the center in centimeters. The difference between the average balance scores (YBT) in each direction was measured separately using Eq. (1). The validity of the YBT was reported to be 0.85–0.93 [29]. Also, there was moderate to high quality evidence demonstrating that the YBT is a reliable dynamic neuromuscular control test with Intra-rater reliability ranged from 0.85 to 0.91 [30]. The normalized average of three repetitions was recorded as the score. The effort was repeated in the event of the following situations: hands being separated from the hip, using the reaching leg to bear weight, moving the supporting leg and losing balance. To calculate the total score of the dynamic balance test, the measurements of all three directions were added together and divided by three times the length of the dominant leg.

$$ \mathbf{S}\mathbf{c}\mathbf{o}\mathbf{r}\mathbf{e}=\frac{\mathbf{r}\mathbf{e}\mathbf{a}\mathbf{c}\mathbf{h} \mathbf{d}\mathbf{i}\mathbf{s}\mathbf{t}\mathbf{a}\mathbf{n}\mathbf{c}\mathbf{e} }{\mathbf{l}\mathbf{e}\mathbf{g} \mathbf{l}\mathbf{e}\mathbf{n}\mathbf{g}\mathbf{t}\mathbf{h}}\times 100$$

### Intervention

#### Knee injury prevention training program (STOP-X program)

Based on the results of medical research, STOP-X has been developed as a prevention concept that can reduce the risk of serious knee joint injuries, which includes programs that can be integrated into normal training. The prevention strategy consisted of several elements, including teaching injury mechanisms, correcting dangerous movement patterns, balance exercises, neuromuscular training for muscular coordination, and hip stabilization exercises [31]. The STOP-X program consisted of running, balance training, a jump-landing pattern, and strength training for 25–40 min for eight weeks (three times per week). Although the program can be used integrally for warm-up, athletes with DKV can run it to correct their defects and reduce the incidence of knee injuries by up to 27% and ACL injuries by up to 51% [11]. The program started with simple exercises, and the difficulty level of individual exercises increased over time. The STOP-X program comprises a variety of proven injury recovery programs, including the Henning program, the Vermont Alpine Knee injury program, the FIFA 11 + program, the Monica Santa Injury program, the Oslo Handball injury program, and the aerial program from German handball injury [31, 32]. The aforementioned training program is presented in Table 1. After the variables were determined during the pretest, the subjects in the experimental group performed the STOP-X program for eight weeks as warm-ups at the beginning of the basketball exercises. In contrast, the control group simply performed routine warm-up, including running, stretching, warm-up with a ball at the beginning of the basketball exercises with the same time schedule. Another point is that the routine warm-up program in the control group lacked strength, balance, stability and performance exercises to have the ability to influence the landing mechanics of the athletes, while the STOP-X training include these items.

**Table 1:** The STOP-X training program

factor	Practice/week	1	2	3,4	5	6,7	8
Running and walking	Easy warm-up	5 M	5 M	5 M	5 M	5 M	5 M
	Running with hip external rotation	2*8R	2*10R	2*12R	2*14R	-	-
Balance	Lunge (R-L)	2*10R	2*10R	-	-	-	-
	Lunge on soft pad (R-L)	-	-	2*10R	2*10R	-	-
	Single leg squat (R-L)	2*10R	2*12R	-	-	-	-
	Single leg squat on soft pad (R-L)	-	-	2*10R	2*12R	-	-
	Single leg squat while holding a medicine ball in hand (R-L)	-	-	2*10R	2*12	-	-
	Single leg squat while holding a medicine ball in hand on soft pad (R-L)	-	-	;-	-	2*10R	2*12R
	Clockwise Lunge on the soft pad	-	-	-	-	2R	3R
	Training with partner: the subject stands on a soft pad with one leg and throws a ball with hand (R-L)	-	-	-	-	2*10R	2*12R
	Training with partner: standing on one leg on the balance hemisphere and trying to upset the balance of the partner (R-L)	-	-	-	-	2*4R	2*6R
strength	Side-steps with a resistance band around the ankles	2*10R	2*12R	-	-	-	-
	Squat with theraband	2*10R	2*12R	-	-	-	-
	Front plank	3*30S	3*40S	-	-	-	-
	Dynamic plank	-	-	3*30S	3*45S	3*60S	3*60S
	Side plank with hip lift (R-L)	2*10R	2*12R	-	-	-	-
	Side plank with leg raise (R-L)	-	-	1*15R	1*20R	1*25R	1*30R
	Box squat	2*10R	2*12R	-	-	-	-
	Deep squat on the box	-	-	2*12R	2*14R	-	-
	Single-leg box squat (R-L)	-	-	-	-	2*8R	2*10R
	Nordic hamstring curl with band	10R	12R	2*10R	2*12R	-	-
	Nordic hamstring curl	-	-	-	-	8R	10R
jumping	Long jump	2*8R	2*10	3*8	3*10	-	-
	Running with lateral jump	-	-	-	-	2*8	2*10
	Running with long jump	-	-	-	-	2*8	2*10
	Tuck jump	-	-	-	-	2*8	2*10

rest between sets = 1:1

rest end of sets = 1:2

(The first number is the amount of time used for training and the second number is the amount of rest)

R = Repeat, S = Seconds, M = Minutes, R-L = Right-Left

### Statistical analysis

To assess the normality of data distribution and homogeneity of variances, Shapiro-Wilk and Levene’s tests were used, respectively. Descriptive statistics were calculated for all variables, and mean, and standard deviation (SD) were reported. Independent samples t-test was applied to compare the demographic characteristics of the two groups. Then, according to the research design, Two-factor ANOVA test 2 (group: experimental, control) × 2 (time: pre-test, post-test) with a group x condition interaction was used to analyze the within and between group evaluation over the eight-week STOP-X training. If a significant interaction effect was found between factors, post hoc analyses (paired t-test) with Bonferroni adjustment for pairwise comparisons were applied. Within-group factor (pre-test to post-test) as the main effect of time and between-group as the main effect of the group were considered. Percentage changes from the pre-test to the post-test were calculated. Effect sizes (ES) using partial eta squared were calculated to increase the analysis power. Effect sizes were classified as small (0.01), moderate (0.06), and large (0.14) [33]. A modified intention to treat analysis based on the complete case method was used. In this method, since one person was randomly removed from each control and experimental group, they were excluded from the study. The analysis was performed only on those who completed the pre-test and post-test. Findings were analyzed at a significance level of 95%, with a statistical significance of (*p* < 0.05), and performed using IBM SPSS software (SPSS, version 26, Chicago; IL).

## Results

After completing the data collection form, the subjects (*n* = 30, age = 15.50 ± 1.52 years, height = 1.62 ± 0.06 cm, weight 55.15 ± 9.65 kg, BMI = 20.69 ± 2.80 kg/m^2^_,_ leg length 85.46 ± 3.69 cm, sports history = 3.73 ± 1.56 years) were purposefully selected and randomly divided into experimental (*n* = 15) and control (*n* = 15) groups. There was no significant difference between the two groups in terms of age (*P* = 0.07), height (*P* = 0.056), weight (*P* = 0.84), body mass index (*P* = 0.46), leg length (*P* = 0.38), or sports history (*P* = 0.06) (Table 2). Pre-test comparisons revealed no significant differences between groups at baseline testing for all variables (*P* > 0.05) (Table 3).

**Table 2:** The demographic characteristics of the subjects (mean ± standard deviation)

Measurement index	Group	Mean ± standard deviation	T	P
Age (years)	control	15.00 ± 1.30	1.87	0.07
	experimental	16.00 ± 1.60		
Height (cm)	control	1.60 ± 0.06	1.99	0.056
	experimental	1.65 ± 0.04		
Weight (kg)	control	54.80 ± 11.39	0.19	0.84
	experimental	55.50 ± 7.95		
BMI (kg/m^2^)	control	21.07 ± 3.32	-0.74	0.46
	experimental	20.30 ± 2.23		
Dominant leg length (cm)	control	84.86 ± 4.42	0.88	0.38
	experimental	86.06 ± 2.81		
Sports history (years)	control	3.20 ± 0.86	1.95	0.06
	experimental	4.26 ± 1.93		

**Table 3:** Results of two-factor ANOVA test to compare the mean of variables

Variable	Group	Pre-test Mean ± SD	Post-test Mean ± SD	F	P value	ES	Main effect of group		Main effect of time		Time* Group Interaction effect	
							P value	ES	P value	ES	P value	ES
SB (Second)	CON	28.06 ± 13.16	30.86 ± 16.06	1.06	0.31	0.03	0.001^*^	0.33	0.001^*^	0.56	0.001^*^	0.42
	EXP	34.20 ± 8.70	54.60 ± 8.95	56.45	0.001^*^	0.66						
DBa (%)	CON	60.14 ± 5.55	60.96 ± 5.59	0.56	0.45	0.02	0.17	0.06	0.001^*^	0.52	0.001^*^	0.42
	EXP	59.73 ± 7.85	67.49 ± 6.02	51.09	0.001^*^	0.64						
DBpm (%)	CON	76.56 ± 7.46	77.45 ± 6.83	0.12	0.72	0.004	0.002^*^	0.28	0.001^*^	0.53	0.001^*^	0.48
	EXP	77.06 ± 12.12	96.43 ± 10.01	58.27	0.001^*^	0.67						
DBpl (%)	CON	78.69 ± 11.97	79.66 ± 10.14	0.17	0.67	0.006	0.005^*^	0.25	0.001^*^	0.50	0.001^*^	0.44
	EXP	82.24 ± 12.85	98.58 ± 8.39	50.39	0.001^*^	0.64						
DBT (%)	CON	71.80 ± 7.45	72.69 ± 6.67	0.40	0.52	0.01	0.004^*^	0.25	0.001^*^	0.68	0.001^*^	0.62
	EXP	73.01 ± 9.34	87.50 ± 6.50	107.57	0.001^*^	0.79						
KVA (Degree)	CON	20.00 ± 3.27	20.41 ± 2.80	0.34	0.56	0.01	0.06	0.11	0.001^*^	0.65	0.001^*^	0.70
	EXP	22.03 ± 3.57	14.46 ± 2.72	119.46	0.001^*^	0.81						

*Significance at the *P* < 0.05 level

SB = Static balance, DBa = Dynamic balance anterior, DBpm = Dynamic balance posterior medial, DBpl = Dynamic balance posterior lateral, DBT = Total dynamic balance, KVA = Knee valgus angle, SD = Standard deviation, ES = Effect size.

Therefore, the results of the Shapiro-Wilks and Levene’s tests confirmed that the data were normally distributed, and the variances were homogeneous (*P* > 0.05). All subjects participated in the pre-test and post-test after 8 weeks.

As per Table 3, repeated measures ANOVA results revealed significant effects of the 8-week STOP-X training. Significant group × time interaction effects were found for the static balance (F = 21.01; *P* = 0.001; ES = 0.42), dynamic balance anterior (F = 20.44; *P* = 0.001; ES = 0.42), dynamic balance posterior medial (F = 26.52; *P* = 0.001; ES = 0.48), dynamic balance posterior lateral (F = 22.28; *P* = 0.001; ES = 0.44), total dynamic balance (F = 47.36; *P* = 0.001; ES = 0.62), and knee valgus angle (F = 66.34; *P* = 0.001; ES = 0.70). Additionally, significant main effects of time were found for the static balance (F = 36.50; *P* = 0.001; ES = 0.56), dynamic balance anterior (F = 31.21; *P* = 0.001; ES = 0.52), dynamic balance posterior medial (F = 31.86; *P* = 0.001; ES = 0.53), dynamic balance posterior lateral (F = 28.28; *P* = 0.001; ES = 0.50), total dynamic balance (F = 60.61; *P* = 0.001; ES = 0.68), and knee valgus angle (F = 53.46; *P* = 0.001; ES = 0.65). The main effect of the group was significant at the static balance (F = 14.02; *P* = 0.001; ES = 0.33), dynamic balance posterior medial (F = 11.22; *P* = 0.002; ES = 0.28), dynamic balance posterior lateral (F = 9.40; *P* = 0.005; ES = 0.25), and total dynamic balance (F = 9.59; *P* = 0.004; ES = 0.25).

Post hoc tests showed significant differences in the static balance (F = 56.45; *P* = 0.001; ES = 0.66, PC=↑59.64%), dynamic balance anterior (F = 51.09; *P* = 0.001; ES = 0.64, PC=↑12.99%), dynamic balance posterior medial (F = 58.27; *P* = 0.001; ES = 0.67, PC=↑25.13%), dynamic balance posterior lateral (F = 50.39; *P* = 0.001; ES = 0.64, PC=↑19.86%), total dynamic balance (F = 107.57; *P* = 0.001; ES = 0.79, PC=↑19.84%), and knee valgus angle (F = 119.46; *P* = 0.001; ES = 0.81, PC= ↓34.36%) in the experimental group compared to the control group. However, there was no significant difference between the pre-test and the post-test in the control group (Table 3).

## Discussion

This study explored the effect of the STOP-X program on the DKV angle and on static and dynamic balance in female basketball players with DKV. After eight weeks of training, there was a significant difference in the knee valgus angle (*P* = 0.001, ES = 0.81) and static (*P* = 0.001, ES = 0.66) and dynamic balance (*P* = 0.001, ES = 0.79) between the control and experimental groups. Baba Goltabar and Norasteh (2022) reported significant differences in the knee valgus angle, knee flexion angle at the end of landing, and static and dynamic balance between soccer players in the control and training groups after eight weeks of the STOP-X program [34]. Eslami et al. (2023) reported that STOP-X exercises were more effective than FIFA11 + exercises at improving DKV and balance among young football players with knee valgus abnormalities [15]. Similarly, Mohammadyari et al. (2023) determined the effect of the STOP-X program on knee valgus and static and dynamic balance in military cadets with DKV defects. The results demonstrated a significant decrease in the knee valgus angle and a significant increase in static and dynamic balance in the training group compared to the control group in the posttest [35], which is consistent with the results of the present study. During landing or cutting movements, the knee valgus position is associated with ACL injuries [3]. Otherwise, ACL injuries in athletes are associated with balance and postural control disorders [36]. Moreover, balance training can reduce maximum (peak) valgus torque and internal rotation during weight-bearing activities [37]. Part of the STOP-X program is performed in pairs on an unstable surface. Therefore, individuals had to maintain balance, trunk control, and lower extremities on a soft pad or balance hemisphere against the perturbations caused by a training opponent. A lack of stability during exercise causes instantaneous changes in neuromuscular muscle-tendon units (MTUs). This challenges the ability to recognize and respond (efferent activities) to achieve balance and perform better movements [38]. Since improved lower extremity proprioception performance is linked to improved dynamic balance performance [39], the STOP-X program strengthens proprioception and leg muscles, enhances muscle coordination, and ultimately improves static and dynamic balance with effect sizes of 66% and 79% through unstable training devices such as balance boards and soft foam [37]. Otherwise, core strength training (CST) helps improve dynamic balance and muscle coordination between the lower and upper extremities [40], reduces muscle imbalances, and prevents lower extremity and knee joint injuries [41]. In this context, Stickler et al. indicated that core strength, determined by the side-plank isometric test, was positively related to knee valgus. A 10% change in core strength normalized to body weight leads to a 1.79° improvement in knee valgus [42]. Among the reasons for the positive effect of the STOP-X program in reducing the knee valgus angle with an effect size of 81% in the present study were CST and strength training to strengthen the hip and core muscles (e.g., front planks, side planks, dynamic planks, single-leg and double-leg squats, and jumping exercises). Additionally, jump training programs have been reported to improve knee valgus during landing, indicating a reduced incidence of ACL injuries in female athletes [43, 44]. Consisting of jumping exercises by avoiding knee valgus motion, the STOP-X program is considered an effective solution for reducing the incidence of ACL injuries by reducing knee valgus loading and expanding the reaction force capability. Otherwise, neuromuscular and biomechanical factors should be considered to prevent noncontact ACL injuries because they are the only factors that can be altered through training. The importance of neuromuscular training (NMT) programs with an emphasis on feedforward mechanisms was discussed in the literature review [45]. Feedforward mechanisms allow anticipated landing, maintaining balance during landing, decelerating, cutting maneuvers, and changing directions and are considered among the most crucial factors in reducing the risk of ACL injury. Therefore, the STOP-X program comprises a set of neuromuscular exercises to improve strength, coordination, and balance; subsequently, patients are advised to avoid knee joint injury-prone positions; and ultimately, movement patterns are endangered. In addition to strengthening basic parameters, this program focuses on ensuring proper movement patterns, particularly avoiding knee valgus motion and encouraging greater knee flexion while landing during jumping and cutting movements. The researcher’s verbal feedback during exercise facilitates the subjects’ learning process and improves movement patterns. Another feature of the STOP-X program is focusing on muscle, joint, and nerve chains instead of focusing locally on one or more muscles. Among the reasons for the effectiveness of this training program are its multidimensionality and focus on several factors (including strength, core stability, plyometrics, balance, and functional exercises), because based on previous studies, applying exercises comprehensively can be more effective than using exercises separately [46–48]. The mechanoreceptors lying in the periarticular structures, including the collateral ligaments of the knee, are responsible for the reflex tension of the lateral and medial muscles of the knee, thus counteracting valgus movements [49]. Therefore, if the muscles around the knee joint do not have proper efficiency such as muscle imbalance and delay in firing, the lower limb is placed in an unfavorable alignment and the joint structures are prone to damage by bearing a lot of stress. Therefore, it is important to identify modifiable risk factors and to create and implement interventions to minimize or eliminate these factors to prevent injury. The internal risk factors of the ACL ligament are divided into two categories modifiable and non-modifiable risk factors [50]. Non-modifiable internal risk factors include anatomical and physiological factors that are biologically stable [50]. However, these modifiable risk factors are neuro-muscular trunk control as well as strength, activation, and co-contraction of the muscles (gluteal, quadriceps, hamstrings) as causes of the incorrect movement pattern of the knee, which can be modified with exercise. Meanwhile evidence suggests that decreased hip abductors, extensors, and external rotators strength are potential factors predisposing to knee valgus during SLL among women [51]. During single-leg motor tasks, more eccentric work is required of the standing leg hip abductors to resist the contralateral pelvic depression and femoral adduction [51]. Besides, gluteal muscles work in contradiction to the dynamic knee valgus movement by hip abduction and external rotation [52]. The hamstring and quadriceps muscles, due to their distal tibial attachments, counteract excessive rotational movements and abduction of the knee [53, 54]. Also, muscular imbalance in medial-to-lateral quadriceps-hamstring co-contraction may contribute to increased knee valgus among recreationally active women [55]. Otherwise, when the body is loaded on one leg, hip abductor muscles work with core muscles such as the quadratus lumborum to keep the position of the pelvis horizontally and prevent excessive adduction of the femur [56]. However, it is necessary to pay attention to the strengthening exercises of these muscles to prevent lower limb injuries and improve the DKV pattern. Since the coactivation of muscles is an important mechanism in the stabilization and motor control of the lower limb, it seems that one of the reasons for the effect of STOP-X training on reducing DKV during SLL is the presence of hip and core muscles strength training in addition to other training components. The condition of this phenomenon is the simultaneous contraction of agonists and antagonists of a given joint [57].

Among the limitations of this study are that the researchers could not evaluate the durability of the STOP-X program among female basketball players or that it is possible to screen a larger number of individuals to perform exercise in a larger sample. Thus, researchers are advised to carry out further studies with larger sample sizes in other age groups to better generalize and evaluate the durability of the training program.

## Conclusion

By identifying athletes at risk, variables such as the knee valgus angle and balance can be improved, and neuromuscular disorders can be reduced to some extent by applying effective training programs. According to the results of the present study, female basketball players with DKV defects are advised to perform the STOP-X program repeatedly during the sports period as an effective program to ensure the elimination or reduction of DKV defects. In addition, this program seems to help reduce risk factors for ACL injuries and thus affect biomechanical and neuromuscular factors associated with ACL injuries.

## Data Availability

The data and materials used to support the findings of this study are available from the corresponding author upon request. (sedaghati@guilan.ac.ir).

## Abbreviations

DKV: Dynamic Knee Valgus
ACL: Anterior Cruciate Ligament
SLL: Single-Leg Landing
YBT: Y-Balance Test
DKG: German Knee Society
pKAM: Probability of A High Knee Abduction Moment
MTUs: Muscle-Tendon Units
CST: Core Strength Training
NMT: Neuromuscular Training
PC: Percentage change
